# Evolution of indicators related to experimentation and use of tobacco products among Brazilian adolescents

**DOI:** 10.1590/1980-549720260049

**Published:** 2026-08-28

**Authors:** Deborah Carvalho Malta, Mariana Gonçalves de Freitas, Marco Antonio Ratzsch de Andreazzi, Juliana Bottoni de Souza, Leticia de Oliveira Cardoso

**Affiliations:** I Universidade Federal de Minas Gerais, Department of Maternal-Child Nursing and Public Health – Belo Horizonte (MG), Brazil.; II Ministério da Saúde, Health Surveillance Secretariat – Brasília (DF), Brazil.; III Instituto Brasileiro de Geografia e Estatística, Coordination of Population and Social Indicators – Rio de Janeiro (RJ), Brazil.; IV Universidade Federal de Minas Gerais – Belo Horizonte (MG), Brazil.; V Ministério da Saúde – Brasília (DF), Brazil.

**Keywords:** Tobacco use, Adolescent health, Health surveys, Brazil

## Abstract

**Objective:**

This study aimed to estimate the prevalence of indicators related to experimentation and use of tobacco products among Brazilian students aged 13–17 years in 2024 and to compare them with values observed in 2015 and 2019 in the country.

**Methods:**

A cross-sectional study was conducted, with prevalence estimates according to sociodemographic variables and temporal analysis of the indicators.

**Results:**

An increase was observed in lifetime experimentation (from 16.8 to 29.6%) and electronic cigarette use in the past 30 days (from 2.8 to 8.7%). Conversely, a reduction was observed in cigarette, hookah, and overall tobacco product use in the past 30 days.

**Conclusion:**

The increase in electronic cigarette use among students highlights the need to maintain their prohibition, expand educational actions, and strengthen surveillance and enforcement measures.

## INTRODUCTION

According to the World Health Organization (WHO), tobacco use is responsible for more than 8 million deaths worldwide^1^. Early exposure to nicotine during adolescence can impair brain development and increase the risk of respiratory diseases^2^. Tobacco use within the past 30 days among adolescents is considered an important predictor of continued tobacco use in adulthood^3^.

In recent decades, stable smoking prevalence among adolescents has been observed in several countries, including Brazil, a trend that has been attributed to tobacco control policies^4^. At the same time, the emergence of new products, such as electronic cigarettes, has posed a significant challenge to public health. Previous national studies have also reported an increase in the use of tobacco products among schoolchildren, reinforcing the need for continuous monitoring of tobacco use patterns^4,5^.

Surveillance of health risk factors among schoolchildren is recommended by the WHO and is conducted internationally through initiatives such as the Health Behavior in School-aged Children study^5^. In Brazil, this surveillance is carried out through the National School Health Survey (*Pesquisa Nacional de Saúde do Escolar* – PeNSE), which is essential for monitoring changes in tobacco use patterns, identifying emerging trends at an early stage, evaluating the impact of tobacco control policies, and informing prevention, health education, and regulatory measures to protect young people^6^.

Accordingly, this study aimed to estimate the prevalence of indicators related to the experimentation and use of tobacco products among Brazilian schoolchildren aged 13–17 years in 2024 and to compare these findings with those observed in 2015 and 2019.

## METHODS

Data from the 2015, 2019, and 2024 PeNSE editions - a cross-sectional study conducted by the Brazilian Institute of Geography and Statistics (*Instituto Brasileiro de Geografia e Estatística* – IBGE) in partnership with the Ministry of Health, will be used. The survey included schoolchildren aged 13–17 years enrolled in public and private schools across Brazil. The sample comprised 10,926 schoolchildren in 2015, 125,123 in 2019, and 118,099 in 2024^4,6^.

This study evaluated indicators of lifetime experimentation and past 30-day use of cigarettes, hookahs, electronic cigarettes, and any tobacco products. Experimentation was defined as self-reported use at least once during the participant's lifetime, whereas current use was defined as use on at least one day during the previous 30 days. Details regarding the survey questions and the categorization of the indicators have been described in a previous publication^6^.

Prevalence estimates and 95% confidence intervals (95%CI) were calculated for the total study population and according to age group (13–15 years; 16–17 years), sex (female and male), and type of school (public and private), with sampling weights applied^6^. Prevalence estimates were also compared across the 2015, 2019, and 2024 survey editions, noting that some indicators could not be compared with those from 2015 because of differences in the questionnaire. All statistical analyses were performed using Stata software.

The PeNSE survey editions were approved by the National Research Ethics Commission of the Ministry of Health under approval no. 1.006.467 (2015), No. 3.249.268 (2019), and No. 6.714.771; CAAE 07508818.5.0000.0008 (2024).

### Data availability statement:

The entire set of data that supports the results of this study was published in an article.

## RESULTS

In 2024, the prevalence of lifetime experimentation of cigarettes, hookahs, and electronic cigarettes was 18.5% (95%CI 17.9–19.2), 16.4% (95%CI 15.4–17.5), and 29.6% (95%CI 28.7–30.6), respectively. The highest prevalences were observed among adolescents aged 16–17 years and students attending public schools. For electronic cigarettes, a higher prevalence was also observed among girls (Table 1).

**Table 1:** Prevalence of experimentation and use of tobacco products among adolescents aged 13–17 years, according to sex, age range, and school administrative status. Brazil, 2024.

Indicators	Total	Sex		Age range		School administrative status	
	% (95%CI)	Male	Female	13 to 15 years	16 and 17 years	Public	Private
		% (95%CI)	% (95%CI)	% (95%CI)	% (95%CI)	% (95%CI)	% (95%CI)
Lifetime cigarette experimentation	18.5 (17.9–19.2)	18.3 (17.5–19.0)	18.8 (17.8–19.7)	14.9 (14.1–15.6)	24.9 (23.8–26.0)	19.8 (19.0–20.6)	11.5 (10.9–12.0)
Cigarette use in the past 30 days	5.5 (5.2–5.9)	5.8 (05.3–06.4)	5.3 (4.8–5.7)	4.6 (4.2–5.1)	7.2 (6.5–7.8)	6.1 (5.6–6.5)	2.8 (2.5–3.1)
Lifetime electronic cigarette experimentation	29.6 (28.7–30.6)	27.5 (26.6–28.5)	31.7 (30.6–32.9)	25.2 (24.1–26.3)	37.5 (36.2–38.8)	30.5 (29.43–31.6)	24.9 (23.9–25.9)
Electronic cigarette use in the past 30 days	8.7 (8.2–9.2)	7.5 (7.1–8.1)	9.8 (9.1–10.5)	7.2 (6.7–7.8)	11.2 (10.4–12.0)	8.9 (8.3–9.4)	7.6 (6.9–8.4)
Lifetime hookah experimentation	16.4 (15.4–17.5)	16.4 (15.3–17.6)	16.5 (15.4–17.6)	13.6 (12.5–14.7)	21.5 (20.2–22.9)	18.2 (17.01–19.5)	6.9 (6.4–7.6)
Hookah use in the past 30 days	3.5 (3.0–4.0)	2.9 (2.5–3.5)	4.0 (3.5–4.6)	2.9 (2.4–3.6)	4.5 (3.8–5.2)	3.9 (3.4–4.5)	1.2 (0.9–1.2)
Use of any tobacco product*	13.9 (13.3–14.7)	13.3 (12.5–14.1)	14.7 (13.8–15.6)	12.0 (11.2–12.9)	17.5 (16.5–18.4)	14.9 (14.1–15.8)	9.0 (8.3–9.8)

* Combination of the variables "cigarette smoking in the past 30 days" and the variables for other tobacco products (electronic cigarettes, hookah, hand-rolled cigarettes, bidis, and other tobacco products).

In the same year, the prevalence of past 30-day use was 5.6% (95%CI 5.2–5.9) for cigarettes, 3.5% (95%CI 3.0–4.0) for hookahs, 8.7% (95%CI 8.2–9.2) for electronic cigarettes, and 13.9% (95%CI 13.3–14.7) for any tobacco product. Overall, the highest prevalences were observed among adolescents aged 16–17 years and students attending public schools. An exception was observed for electronic cigarette use, which was more prevalent among girls and adolescents aged 16–17 years (Table 1).

**Figure 1:**
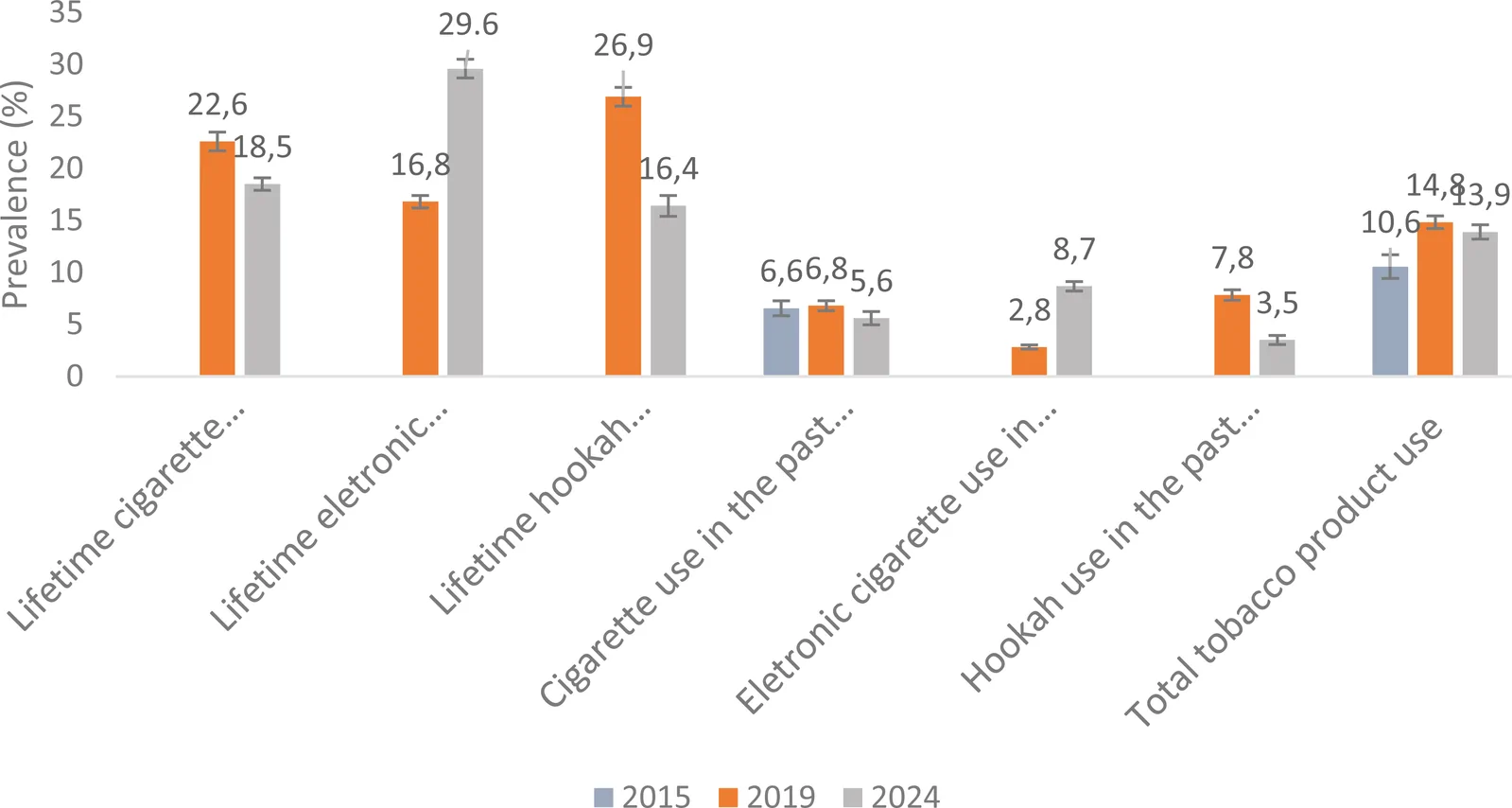
Prevalence of experimentation and use of tobacco products among adolescents aged 13–17 years in 2015*, 2019, and 2024. Brazil, 2024.

Temporal analysis of the PeNSE survey editions showed a decline in lifetime experimentation with cigarettes, from 22.6 in 2019 to 18.5% in 2024, and with hookahs, from 26.9 to 16.4% over the same period. In contrast, lifetime experimentation with electronic cigarettes increased from 16.8 in 2019 to 29.6% in 2024 (Figure 1).

*In 2015, due to the use of different questionnaires, not all indicators are comparable; therefore, some were not presented; "Experimentation" refers to lifetime experimentation.

Past 30-day cigarette use remained relatively stable between 2015 and 2019, increasing slightly from 6.6% (95%CI 5.8–7.3) to 6.8% (95%CI 6.3–7.3), followed by a decline in 2024. Past 30-day hookah use also decreased between 2019 and 2024, from 7.8% (95%CI 7.3–8.3) to 3.5%. In contrast, past 30-day electronic cigarette use increased markedly, from 2.8% (95%CI 2.6–3.0) in 2019 to 8.7% in 2024. The prevalence of past 30-day use of any tobacco product increased between 2015 and 2019, from 10.6 to 14.8%, followed by a significant decline in 2024, reaching 13.9% (Figure 1).

## DISCUSSION

Compared with the 2015 and 2019 PeNSE survey editions, reductions in both lifetime experimentation and recent use of cigarettes, hookahs, and any tobacco products were observed among Brazilian adolescents in 2024, suggesting a possible positive impact of the tobacco control policies implemented in the country. In contrast, lifetime experimentation with electronic cigarettes was reported by approximately one-third of schoolchildren, and past 30-day use quadrupled between 2019 and 2024, reaching 8.7% of adolescents.

Similar trends have been observed internationally, with a decline in conventional tobacco use among adolescents since 2018 and an increase in electronic cigarette use, particularly among young people^5^. This increase has been associated with digital advertising, the wide variety of available flavors, the perception of reduced health risks, peer and social media influence, and ease of access, making electronic cigarette use a major public health concern^1,2,4,5^.

The WHO estimates that 18% of adolescents have experimented with electronic cigarettes and that 10% have used them within the past 30 days^5^. In the present study, the prevalence of lifetime experimentation exceeded the global estimate, whereas the prevalence of past 30-day use was of a similar magnitude. Consistent with the international literature, a higher prevalence was also observed among girls and older adolescents^5,6^.

Despite the ban on the sale of electronic cigarettes in Brazil since 2009, their use has increased among adolescents. Strengthening regulatory, enforcement, and health education initiatives, in addition to expanding the continuous monitoring of tobacco use patterns, is essential to prevent early initiation and reduce the impact of nicotine use among young people^1,2,4^.

## References

[1] Organização Pan-Americana da Saúde (OPAS) Tabaco [Internet].

[2] World Health Organization (WHO) (2019). WHO Report on the Global Tobacco Epidemic, 2019.

[3] Dutra LM, Glantz SA (2018). Smoking in the last thirty days during adolescence is a strong predictor of smoking in young adulthood. Prev Med.

[4] Malta DC, Morais EAH, Silva AG, Souza JB, Gomes CS, Santos FM (2024). Mudanças no uso do tabaco entre adolescentes brasileiros e fatores associados: Pesquisa Nacional de Saúde do Escolar. Ciênc Saúde Coletiva.

[5] Charrier L, van Dorsselaer S, Canale N, Baska T, Kilibarda B, Comoretto RI, World Health Organization (WHO) (2024). Health behaviour in school-aged children international report from the 2021/2022 survey.

[6] Instituto Brasileiro de Geografia e Estatística (IBGE) (2026). Pesquisa Nacional de Saúde do Escolar (PeNSE): 2024.

