# Supplementary material for: Evolution of indicators related to experimentation and use of tobacco products among Brazilian adolescents
Source: Rev Bras Epidemiol. 2026 Aug 28;29:e260049. doi: 10.1590/1980-549720260049 (PMC13524306; doi:10.1590/1980-549720260049)
Supplement: Translation

# Translation: Evolução dos indicadores relacionados à experimentação e ao uso de produtos derivados do tabaco entre adolescentes brasileiros

**DOI:** 10.1590/1980-549720260049.2

**Authors:** Deborah Carvalho Malta, Mariana Gonçalves de Freitas, Marco Antonio Ratzsch de Andreazzi, Juliana Bottoni de Souza, Leticia de Oliveira Cardoso

**Affiliations:** I Universidade Federal de Minas Gerais, Departamento de Enfermagem Materno-Infantil e Saúde Pública – Belo Horizonte (MG), Brasil.; II Ministério da Saúde, Secretaria de Vigilância em Saúde – Brasília (DF), Brasil.; III Instituto Brasileiro de Geografia e Estatística, Coordenação de População e Indicadores Sociais – Rio de Janeiro (RJ), Brasil.; IV Universidade Federal de Minas Gerais – Belo Horizonte (MG), Brasil.; V Ministério da Saúde – Brasília (DF), Brasil.

**Keywords:** Uso de tabaco, Saúde do adolescente, Inquéritos epidemiológicos, Brasil

## Abstract

**Objetivo:**

O estudo teve como objetivo estimar as prevalências de indicadores relacionados à experimentação e ao uso de produtos derivados do tabaco entre escolares de 13 a 17 anos de idade brasileiros em 2024 e compará-las aos valores observados em 2015 e 2019 no país.

**Métodos:**

Estudo transversal, com cálculo das prevalências segundo variáveis sociodemográficas e análise temporal dos indicadores.

**Resultados:**

Observou-se aumento da experimentação na vida (16,8% para 29,6%) e no uso de cigarro eletrônico nos últimos 30 dias (2,8% para 8,7%). Em contrapartida, houve redução do uso de cigarros, narguilé e de todos os produtos do tabaco nos últimos 30 dias.

**Conclusão:**

Conclui-se que o crescimento do uso de cigarros eletrônicos entre escolares reforça a necessidade de manter sua proibição, ampliar ações educativas e fortalecer a fiscalização.

## INTRODUÇÃO

Segundo a Organização Mundial da Saúde (OMS), o tabaco é responsável por mais de 8 milhões de mortes globalmente^1^. A exposição precoce à nicotina na adolescência pode comprometer o desenvolvimento cerebral e aumentar o risco de doenças respiratórias^2^. O consumo de tabaco nos últimos 30 dias entre adolescentes é considerado importante preditor da manutenção do hábito em adultos^3^.

Nas últimas décadas, observou-se estabilidade do tabagismo entre adolescentes em diversos países, incluindo o Brasil, resultado associado às políticas de controle da substância^4^. Entretanto, o surgimento de novos produtos, como cigarros eletrônicos, tem representado importante desafio para a saúde pública. Estudos nacionais anteriores já evidenciaram aumento do consumo de produtos do tabaco entre escolares, reforçando a necessidade de monitoramento contínuo desse comportamento^4,5^.

A vigilância dos fatores de risco à saúde entre escolares é recomendada pela OMS e realizada internacionalmente por inquéritos como o *Health Behaviour in School-aged Children* ^5^. No Brasil, esse monitoramento ocorre por meio da Pesquisa Nacional de Saúde do Escolar (PeNSE), sendo fundamental para acompanhar mudanças nos padrões de consumo, identificar precocemente novas tendências, avaliar o impacto das políticas de controle do tabaco e subsidiar ações de prevenção, educação em saúde e regulação voltadas à proteção dos jovens^6^.

Assim, o presente estudo objetiva estimar as prevalências de indicadores relacionados à experimentação e ao uso de produtos derivados do tabaco entre escolares de 13 a 17 anos de idade brasileiros em 2024 e compará-las aos valores observados em 2015 e 2019 no país.

## MÉTODOS

Utilizaram-se dados das edições da PeNSE de 2015, 2019 e 2024 - estudo transversal realizado pelo Instituto Brasileiro de Geografia e Estatística (IBGE), em parceria com o Ministério da Saúde, com escolares de 13 a 17 anos matriculados em escolas públicas e privadas do Brasil. A amostra foi composta por 10.926 escolares em 2015, 125.123 em 2019 e 118.099 em 2024^4,6^.

O presente estudo avaliou indicadores de experimentação na vida e uso nos últimos 30 dias de cigarro, narguilé, cigarro eletrônico e qualquer produto do tabaco. A experimentação foi definida pelo relato de uso ao menos uma vez na vida, e o uso atual pelo consumo em pelo menos um dia nos últimos 30 dias. Detalhes sobre as perguntas e a categorização dos indicadores podem ser encontrados em publicação anterior^6^.

Foram estimados prevalências e Intervalos de Confiança de 95% (IC95%) dos indicadores para população total e segundo faixa etária (13 a 15 anos; 16 a 17 anos), sexo (feminino e masculino) e tipo de escola (pública e privada), e aplicados pesos amostrais^6^. Também foram comparadas as prevalências entre as edições de 2015, 2019 e 2024, destacando-se que alguns indicadores não são comparáveis em 2015 devido a diferenças no questionário. Todas as análises foram realizadas utilizando o software *Stata*.

As edições da PeNSE foram aprovadas pela Comissão Nacional de Ética em Pesquisa do Ministério da Saúde, sob os pareceres n.° 1.006.467 (2015), n.° 3.249.268 (2019) e n.° 6.714.771; CAAE 07508818.5.0000.0008 (2024).

### Declaração de disponibilidade de dados:

Todo o conjunto de dados que dá suporte aos resultados deste estudo foi publicado no artigo.

## RESULTADOS

Em 2024, a experimentação na vida de cigarro, narguilé e cigarro eletrônico foi de 18,5% (IC95% 17,9–19,2), 16,4% (IC95% 15,4–17,5) e 29,6% (IC95% 28,7–30,6), respectivamente. As maiores prevalências foram observadas entre adolescentes de 16 a 17 anos e estudantes de escolas públicas; para cigarro eletrônico, também se observou maior frequência entre meninas (Tabela 1).

**Tabela 1:** Prevalência de experimentação e uso de produtos do tabaco entre adolescentes de 13 a 17 anos, segundo sexo, faixa etária e dependência administrativa da escola. Brasil, 2024.

Indicadores	Total	Sexo		Grupo etário		Dependência administrativa da escola	
	% (IC95%)	Masculino	Feminino	13 a 15 anos	16 e 17 anos	Pública	Privada
		% (IC95%)	% (IC95%)	% (IC95%)	% (IC95%)	% (IC95%)	% (IC95%)
Experimentação na vida de cigarro	18,5 (17,9–19,2)	18,3 (17,5–19,0)	18,8 (17,8–19,7)	14,9 (14,1–15,6)	24,9 (23,8–26,0)	19,8 (19,0–20,6)	11,5 (10,9–12,0)
Uso de cigarro nos últimos 30 dias	5,5 (5,2–5,9)	5,8 (05,3–06,4)	5,3 (4,8–5,7)	4,6 (4,2–5,1)	7,2 (6,5–7,8)	6,1 (5,6–6,5)	2,8 (2,5–3,1)
Experimentação na vida de cigarro eletrônico	29,6 (28,7–30,6)	27,5 (26,6–28,5)	31,7 (30,6–32,9)	25,2 (24,1–26,3)	37,5 (36,2–38,8)	30,5 (29,43–31,6)	24,9 (23,9–25,9)
Uso de cigarro eletrônico nos últimos 30 dias	8,7 (8,2–9,2)	7,5 (7,1–8,1)	9,8 (9,1–10,5)	7,2 (6,7–7,8)	11,2 (10,4–12,0)	8,9 (8,3–9,4)	7,6 (6,9–8,4)
Experimentação na vida de narguilé	16,4 (15,4–17,5)	16,4 (15,3–17,6)	16,5 (15,4–17,6)	13,6 (12,5–14,7)	21,5 (20,2–22,9)	18,2 (17,01–19,5)	6,9 (6,4–7,6)
Uso de narguilé nos últimos 30 dias	3,5 (3,0–4,0)	2,9 (2,5–3,5)	4,0 (3,5–4,6)	2,9 (2,4–3,6)	4,5 (3,8–5,2)	3,9 (3,4–4,5)	1,2 (0,9–1,2)
Uso de qualquer produto do tabaco*	13,9 (13,3–14,7)	13,3 (12,5–14,1)	14,7 (13,8–15,6)	12,0 (11,2–12,9)	17,5 (16,5–18,4)	14,9 (14,1–15,8)	9,0 (8,3–9,8)

* Junção das variáveis "uso do fumo nos 30 dias" e das variáveis dos outros produtos do tabaco (cigarro eletrônico, narguilé, cigarro de palha, cigarro de Bali e outros).

No mesmo ano, o uso nos últimos 30 dias foi de 5,6% (IC95% 5,2–5,9) para cigarro, 3,5% (IC95% 3,0–4,0) para narguilé, 8,7% (IC95% 8,2–9,2) para cigarro eletrônico e 13,9% (IC95% 13,3–14,7) para qualquer produto do tabaco. De modo geral, as maiores prevalências ocorreram entre adolescentes de 16 a 17 anos e estudantes de escolas públicas, exceto para o uso de cigarro eletrônico, que foi mais frequente entre meninas e adolescentes de 16 a 17 anos (Tabela 1).

Na análise temporal das edições da PeNSE, observou-se redução da experimentação na vida de cigarro, de 22,6 em 2019 para 18,5% em 2024, e de narguilé, de 26,9 para 16,4% no mesmo período. Em contrapartida, a experimentação de cigarro eletrônico aumentou de 16,8 em 2019 para 29,6% em 2024 (Figura 1).

**Figura 1:** Prevalência de experimentação e uso de produtos do tabaco entre adolescentes de 13 a 17 anos, em 2015*, 2019 e 2024. Brasil, 2024.

*Em 2015, devido ao uso de questionários distintos, nem todos os indicadores são comparáveis, razão pela qual alguns não foram apresentados; "Experimentação" refere-se à experimentação na vida.

Quanto ao uso nos últimos 30 dias, o de cigarro manteve-se relativamente estável entre 2015 e 2019, passando de 6,6% (IC95% 5,8–7,3) para 6,8% (IC95% 6,3–7,3), seguido de redução em 2024. O uso de narguilé também diminuiu entre 2019 e 2024, de 7,8% (IC95% 7,3–8,3) para 3,5%. Já o uso de cigarro eletrônico apresentou aumento expressivo, de 2,8% (IC95% 2,6–3,0) em 2019 para 8,7% em 2024. O uso de qualquer produto do tabaco aumentou entre 2015 e 2019, de 10,6 para 14,8%, mas apresentou redução em 2024, atingindo 13,9% (Figura 1).

## DISCUSSÃO

Em comparação às edições de 2015 e 2019 da PeNSE, observou-se redução da experimentação e do uso recente de cigarro, narguilé e de qualquer produto do tabaco entre adolescentes brasileiros em 2024, sugerindo possível impacto positivo das políticas de controle do tabaco implementadas no país. Em sentido oposto, a experimentação de cigarros eletrônicos atingiu cerca de um terço dos escolares, e o uso nos últimos 30 dias quadruplicou entre 2019 e 2024, alcançando 8,7% dos adolescentes.

Achados semelhantes têm sido observados internacionalmente, com redução do consumo de tabaco convencional entre adolescentes desde 2018 e aumento do uso de cigarros eletrônicos, especialmente entre jovens^5^. Esse crescimento tem sido associado à publicidade digital, variedade de sabores, percepção de menor risco, influência de pares e redes sociais, além da facilidade de acesso, configurando-se como uma importante preocupação de saúde pública^1,2,4,5^.

A OMS estimou que 18% dos adolescentes relataram experimentação de cigarros eletrônicos e 10%, uso nos últimos 30 dias^5^. Nesse estudo, a experimentação foi superior à estimativa global, enquanto o uso recente apresentou magnitude semelhante. Também em consonância com a literatura internacional, observou-se maior prevalência entre meninas e adolescentes mais velhos^5,6^.

Apesar da proibição da comercialização de cigarros eletrônicos no Brasil desde 2009, seu consumo aumentou entre adolescentes. Torna-se fundamental fortalecer ações regulatórias, de fiscalização e de educação em saúde, além de ampliar o monitoramento contínuo desse comportamento, visando prevenir a iniciação precoce e reduzir os impactos do uso de nicotina na população jovem^1,2,4^.
